# Nutrition and Golf Performance: A Systematic Scoping Review

**DOI:** 10.1007/s40279-024-02095-0

**Published:** 2024-09-30

**Authors:** Amy O’Donnell, Andrew Murray, Alice Nguyen, Thomas Salmon, Sam Taylor, James P. Morton, Graeme L. Close

**Affiliations:** 1 Research Institute for Sport and Exercise Sciences, Liverpool John Moores University, Liverpool, L3 3AF UK; 2 Ladies European Tour Performance Institute, Denham, UK; 3 Medical and Scientific Department, The R&A, St Andrews, UK; 4 PGA European Tour Health and Performance Institute, Virginia Water, UK

## Abstract

**Background:**

Golf is played both recreationally and professionally by approximately 66.6 million people worldwide. Despite the potential for nutrition to influence golf performance, research in this area is somewhat limited.

**Objective:**

To identify the existing literature regarding nutrition and golf and where the current research gaps lie.

**Design:**

Scoping review. Online databases were used to retrieve data from 2003 to the present day.

**Data Sources:**

A three-step search strategy identified relevant primary and secondary articles as well as grey literature. Published and unpublished articles in the English language, identified by searching electronic databases (ProQuest Central, Web of Science, Scopus, SPORTDiscus and PubMed) and reference searching.

**Review Methods:**

Relevant identified studies were screened for final inclusion. Data were extracted using a standardised tool to create a descriptive analysis and a thematic summary. In summary, studies were included if they focused on nutrition, hydration, energy requirements, supplements, or body composition in relation to golf.

**Results and Discussion:**

Our initial search found 3616 relevant articles. Eighty-two of these articles were included for the scoping review. Nutrition has the potential to impact golf performance in areas including the maintenance of energy levels, cognitive function, and body composition. Currently, there is limited research available discussing the effects of nutrition interventions related specifically to golf performance.

**Conclusion:**

This scoping review highlights that more work is needed to provide golfers and practitioners with golf-specific nutrition research. The key areas for future golf-specific nutrition research include nutrition on cognitive performance, body composition, energy requirements, supplementation, and the potential role of nutrition for the travelling golfer. Systematic reviews could also be used to identify future priorities for nutrition and golf research.

**Supplementary Information:**

The online version contains supplementary material available at 10.1007/s40279-024-02095-0.

## Key Points

**Table:** 

*Impact of nutrition on golf* Nutrition significantly influences golf performance by affecting energy levels, cognitive function, and body composition, yet specific research on these effects in golf is limited.
*Current research gaps* Eighty-two articles from 16 countries were identified as relevant to this scoping review. There was a lack of comprehensive studies on the effects of nutrition interventions specifically tailored for golfers, highlighting a need for more targeted research in this area.
*Future research directions* Key areas for future research include exploring the role of nutrition in cognitive performance, body composition, energy requirements, supplementation, and addressing the nutritional needs of travelling golfers.

## Introduction

Golf is a sport played recreationally and professionally, by almost all ages, with an estimated 66.6 million people participating globally [1]. Golf is played over courses which differ in distance, terrains and climates, with an 18-hole game (“round”) lasting anywhere between 4 and 6 h [2], not including additional practice and other aspects of performance training such as resistance training. The most recent literature suggests that golf can provide a high-volume, moderate-intensity activity [~ 4.8 metabolic equivalents (METs)] with numerous physical and mental health benefits associated with playing the sport [3, 4]. Poor nutrition and hydration may adversely affect golf performance, from both a physical and cognitive perspective [5, 6]; however, most of the published research in golf has focussed on aspects of general health, strength and conditioning, or biomechanics [4, 7, 8], with limited high-quality studies on the nutritional requirements of the sport.

The physical and lifestyle demands of a touring professional golfer are extensive, suggesting that targeted nutrition support could be of benefit in terms of the general health and the on-course performance of the players. For example, during the 2024 DP World Tour Season, a typical golfer has the option to compete in 45 events in 24 countries across five continents**.** The Ladies European Tour follows a similar schedule (30 events, 18 countries, five continents). Typically, tournaments last for 4 days, with 18 holes a day, before a cut after the second round. Globally, there are also an estimated > 66 million amateur golfers who may also want to maximise their health and performance through improving their nutrition and hydration strategies both on and off the golf course [9].

As a result of the unique demands of golf, it is important to understand the day-day nutritional and energetic demands of golfers as well as researching the impacts that the demanding golfing schedule of the professional player may have. Although not specifically studied in golf, research suggests that nutrition strategies can be used to ensure golfers fuel themselves correctly for performance, recovery and overall health, reducing the potential effects of jet lag and travel fatigue, and mitigating illness such as upper tract respiratory infections [10].

Whilst review articles note a relative dearth of relevant literature, to date there have been no reviews using rigorous methods to search systematically for available articles, summarise this information, and identify knowledge gaps. Therefore, the aim of this scoping review was to explore the current literature on nutrition, hydration, dietary supplements, and the energy requirements of golf, and to identify gaps in the existing research. This information will help to direct future research in golf and identify areas that require immediate attention.

## Methods

The scoping review followed the well-established five stages as suggested by Arksey and O’Malley [11], integrating suggested enhancements from Levac et al. [12] and the Joanna Briggs Institute [13]. Below we summarise our approach to each stage of the scoping review process.

### Stage 1: Identification of the Research Question

Considering the population, context and topics of interest, a broad research question was decided upon:

“Where does the current research lie with regards to nutrition, hydration, supplement and energy requirements and its relationship with golf and performance?”

### Stage 2: Identification of Relevant Studies

The following inclusion and exclusion criteria were established through discussion and consultation between the author group and a university librarian.

#### Inclusion Criteria

- All age groups and both sexes of participants.
- Research articles not limited by geographical location or setting.
- Published in English language.
- Full text links available.
- Any levels of golf (e.g., recreational, amateur, professional, elite).
- Any physical and/or mental health condition.
- All forms of golf (including but not limited to 18 holes, 9 holes, driving range, simulated play) and all methods of transportation of clubs (including but not limited to carrying the bag, using an electric trolley, using a pull trolley, using a buggy, and using a caddy).
- Research published in the last 20 years from the last search date: 21 December 2003 onwards. Search period: October 2023–21 December 2023.
- Sources of information—including primary and secondary research studies, reviews, systematic reviews, scoping reviews, case studies, meta-analyses, guidelines, as well as grey literature to include unpublished and ongoing trials, annual reports, dissertations, and conference proceedings.

#### Exclusion Criteria

- Research regarding caddies.
- Opinion pieces/opinions, magazines, newspaper articles, paper with no data, trade journals, wire feeds, reports with no data.
- Studies focussing on biomechanics or psychology.

### Search Strategies and Databases

The search strategy aimed to discover both published and unpublished studies. An initial limited search of ProQuest Central and Web of Science was conducted to identify relevant articles on the topic. The text words contained in the titles and abstracts of applicable articles, and the index terms used to describe the articles, were used to develop a full search strategy for ProQuest Central, Web of Science, Scopus, SPORTDiscus and PubMed (see Appendix 1 in the Online Supplemental Material for full search terms and search history). Boolean terms ‘AND’ and ‘OR’ were used to extract relevant literature. The search strategy, including all identified keywords and index terms, was adapted for each database mentioned. The reference lists of all included studies was screened for additional research.

### Stage 3: Study Selection

**Fig. 1:**
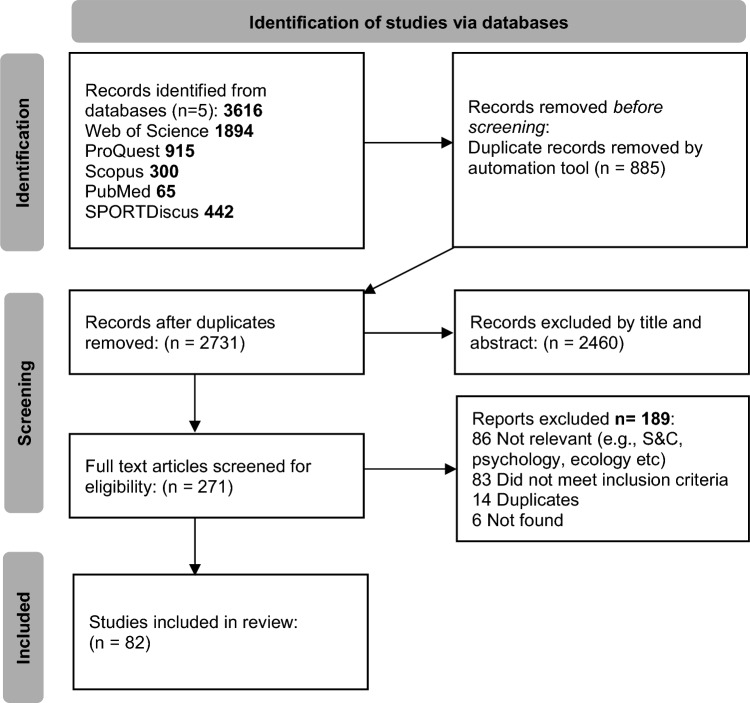
PRISMA-ScR flow chart of the included literature. *S&C* strength and conditioning

Following the search, all identified citations were collated and uploaded into Endnote 21, Web Version (Clarivate Analytics, PA, USA) and duplicates removed. Titles and abstracts were then screened by four lead independent reviewers (AOD, AN, ST, TS) for assessment against the inclusion and exclusion criteria. Potentially relevant sources were retrieved in full, and their citation details imported into Endnote 21, Web Version (Clarivate Analytics, PA, USA). The full set of selected literature was assessed in detail by the lead reviewer (AOD) against the inclusion and exclusion criteria. Secondary reviewers (AN, ST, TS) each completed the same process on a random sample of 10% of the titles with concordance > 97%. Where a decision was not reached at any stage of the selection process, it was resolved through discussion or with an additional reviewer. The results of this search and study inclusion process are reported in full in the final scoping review and are presented in a Preferred Reporting Items for Systematic Reviews and Meta-Analyses extension for scoping review (PRISMA-ScR) flow diagram (Fig. 1) [14].

### Stage 4: Charting the Data

Charting tables to record and collect extracted data from included studies were developed. Data were extracted from papers included in the scoping review by the four independent reviewers (AOD, AN, ST, TS). The lead reviewer (AOD) extracted 90% of the data and secondary reviewers (AN, ST, TS) extracted the remaining 10% of data from the included studies. Each reviewer checked 10% of each other’s data extractions for accuracy.

The draft data extraction tool was modified and revised as necessary during the process of extracting data from each included evidence source. Any discrepancies were discussed at group meetings, and, where necessary, an additional reviewer (GC) was consulted. Concordance was > 97% regarding inclusion/ exclusion. If necessary, authors were contacted to request additional or missing data.

Data extraction categories included: author, year of publication, where the study was published/conducted, aims/purpose, study population and sample size (if applicable), methodology/ intervention type, outcomes and details of these (e.g., how measured) and key findings that related to the scoping review research questions.

### Stage 5: Collating, Summarising and Reporting the Results

Methods undertaken in the protocol by Murray et al. [15] permitted us to collate existing knowledge on this topic and report as:

1. A descriptive analysis, mapping the data, showing distribution of the studies by publication period, country of origin, study method and theme.
2. A thematic analysis, characterising how identified literature relates to the research question and aims, and the main findings from these organised by theme.

In this scoping review, we aimed to (i) map the evidence and key concepts available for golf, nutrition, hydration, and energy requirements; (ii) report and summarise existing research findings for players, practitioners, and relevant stakeholders; and (iii) identify gaps in the existing literature to guide future research directions.

## Results and Discussion

### Descriptive Analysis

A (PRISMA-ScR) flow diagram was produced to report the results from the search and study selection process (Fig. 1). The first search found 3616 relevant articles from the selected databases. Of these articles, 2731 articles were identified once duplicate records were removed by an automated tool (Endnote, Clarivate Analytics, PA, USA). After records had been excluded based on their title and abstract, a further 2460 records were removed, and 271 articles remained. A further six articles were excluded as the full text was not available, despite searching the Liverpool John Moores University Library databases and using interlibrary loans. Therefore, 265 articles underwent full text screening. In total, the scoping review identified 82 eligible articles, including nine from grey literature. The included literature was relevant to the aims and research question, “Where does the current research lie with regards to nutrition, hydration, energy requirements and its effects on golf and performance”, and these were included in the analysis.

### Characteristics of Studies

#### Geography of Included Studies

Research was found from 16 countries (Table 1). The greatest number of studies took place in the USA (41.5%), followed by the UK (20.7%), Europe (17.1%) and Australia (7.3%), which broadly aligns with the regions where golf is most played [1].

**Table 1:** Geography of included studies

Country	No. of studies	Percentage of studies
USA	34	41.5
UK	17	20.7
Australia	6	7.3
Germany, Korea	4 each	4.9 each
Japan	3	3.7
Canada, Lithuania, Turkiye, Spain	2 each	2.4 each
Austria, Finland, Italy, New Zealand, South Africa, Sweden	1 each	1.2 each
All	82	100%

#### Study Design

**Fig. 2:**
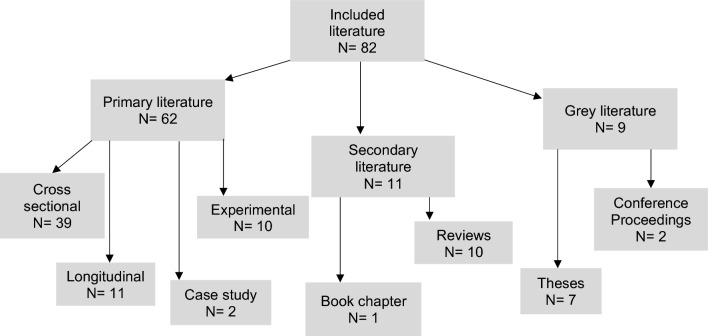
Taxonomy of research designs of included literature

The research included in this scoping review varied in terms of study design, participants and focus. No formal quality assessment of the included literature was undertaken as the aim of a scoping review is to provide a broad picture of the available evidence [13]. Of the 82 studies included, 62 (75.6%) were considered primary literature, 11 (13.4%) secondary, and nine (11.0%) grey literature. A taxonomy of the research included by this scoping review is shown in Fig. 2. Of the primary literature, 39 (62.9%) studies had a cross-sectional design, 11 (17.7%) a longitudinal design, and ten (16.1%) were of experimental design that mainly reported physiological measures such as calories expended. Two (3.2%) were case studies. The secondary studies consisted of reviews (systematic, narrative and one scoping review) and one relevant book chapter was included. These reviews focussed mainly on general nutrition and hydration recommendations based upon guidelines from other sports given the lack of golf-specific studies to review. Of the nine grey literature sources included, seven of these were research theses and two were published conference proceedings.

#### Theme of the Scoping Review

**Fig. 3:**
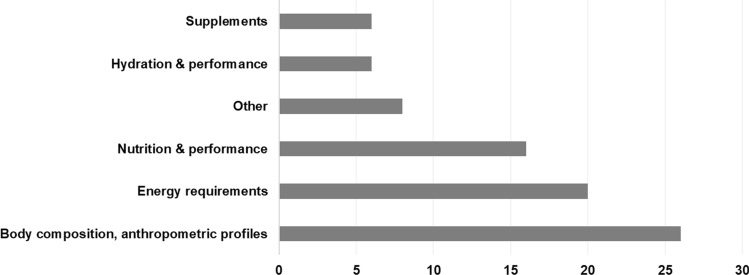
Main themes of the scoping review

The focus of the studies presented was placed into five evident themes (Fig. 3) from the data extraction categories used: (i) nutrition and golf performance (*N* = 16); (ii) hydration and golf performance (*N* = 6); (iii) energy requirements and golf (*N* = 20); (iv) supplements and golf performance (*N* = 6); and body composition, anthropometric profiles and golf performance (*N* = 26).

Research focusing on body composition and anthropometric profiles was the most frequent (31.7%). Further themes included ‘nutrition for travel’ and ‘nutrition for cognitive performance’, and were grouped into category ‘other and general’ (*N* = 8).

## Thematic Summary

### Key Concepts and Evidence Available

#### Macronutrient Requirements for Golf Performance

Golf generally provides moderate aerobic physical activity with an average MET value of 4.5 METs [4]. Golf can also provide low- or high-intensity aerobic exercise depending on age and fitness of participants, the layout of the course, and whether a golf cart is being ridden. The typical distance walked during a round of golf can vary dependent on a number of factors (e.g., number of holes played, length of the course, scoring ability), but it has been reported that golfers can potentially cover in excess of 10 km during a round, with a round typically lasting between 4 and 6 h [16]. Golfers may experience fatigue (both mentally and physically) in the latter stages of a round. The fatigue experienced during a round could be partly attributed to the decline in blood glucose, which has been reported to be as much as 10–30% after an 18-hole game when no nutrition was consumed [17]. Golf-specific carbohydrate research has suggested that the consumption of carbohydrates or carbohydrates with protein, may alleviate perceived levels of fatigue and improve physical performance (assessed via driving accuracy of six amateurs with an average handicap (HCP) index of 8.5 ± 6.72 throughout the first 9 holes) [18]. Although blood glucose was not measured in this study, further research fed 12 competitive amateurs carbohydrate in the form of gummies (30 g/h) throughout 18 holes whilst measuring interstitial glucose concentration and anticipated feelings of fatigue [19]. These authors reported that falls in interstitial glucose and anticipated fatigue were significantly reduced, with main effects for trial and time for interstitial glucose (trial:* p* < 0.001, time: *p* < 0.001; interaction: *p* = 0.923) in the carbohydrate fed group compared with the control group. Interstitial glucose concentrations were significantly higher in the carbohydrate group at the 4th–6th (*p* = 0.042); 7th–9th (*p* = 0.046); 10th–12th (*p* = 0.037); 13th–15th (*p* = 0.020), and 16th–18th holes (*p* = 0.023), suggesting that carbohydrate feeding may help maintain performance during golf [19].

In an MSc thesis by Robinson [20], low glycaemic index (LGI) and high glycaemic index (HGI) carbohydrates were provided to six amateur golfers (HCP Index ≤ 15.4) before and throughout competitive golf play. There were no significant differences in blood glucose at any time point between the LGI and HGI groups, with decreases in blood glucose of 0.3 mmol/L observed when measured at the 10th hole, and a similar decrease when measured again at the 18th hole. The total decrease in blood glucose was ~ 10% in both conditions. There was no significant difference in golf-related performance metrics between the two groups, suggesting that the GI of the carbohydrate, when consumed pre and during competition, does not affect golfing performance or blood glucose concentrations [20].

Outside of the on-course golf demands, it is also important to consider the overall carbohydrate requirements of golfers based upon their baseline requirements and additional training demands. It is now widely recognised that increasing muscle strength has the potential to improve golf performance [21–23] and therefore many golfers incorporate resistance training into their daily schedule. Whist there are guidelines recommending carbohydrate intakes between 3 and 5 g/kg body mass (BM) a day for low-intensity or skill-based activity [24], to date there are no specific recommendations from peer-reviewed literature in terms of the carbohydrate requirements for amateur or professional golfers taking into consideration the on-course, off-course and, for some, travel demands.

Given the importance of dietary protein in muscle protein synthesis, and the growing interest in increasing muscle size and strength in golfers, it was somewhat surprising that there were no relevant studies on the protein requirements for golf. One study examined the effects of protein ingestion when combined with carbohydrate prior to a 9-hole round on acute performance by assessing driving distance, and accuracy of driving, iron shots, chipping and putting [18]. There were significantly lower perceived levels of fatigue when consuming the combined protein and carbohydrate feeding compared with both the placebo and carbohydrate alone, highlighting that a mixed meal may be more beneficial consumed together during golfing performance. Future research must now address the protein requirements for golfers in terms of the timing, type and total amounts required for the long-term development of the golfer especially considering that golfers often play on consecutive days and increasing dietary protein may support muscle recovery and repair.

There were no published studies on the effects of fat consumption on either acute golf performance or long-term health. Considering a round of golf can last anywhere from 4 to 6 h, and is generally of moderate intensity for amateurs and low intensity for professionals (who are typically younger and do not carry their own bags), golf may be a sport where it is possible to fuel the performance using fat as a fuel source [25]. Future research should attempt to identify if it is possible to become fat adapted for golf and if this is an optimal strategy to fuel on-course performance and support improvements in long term health.

#### Hydration and Golf

Hydration on the golf course can be affected by several factors including participant demographics, fluid intake, exercise intensity, sweat rate, competition climate and the type of clothing worn. Dehydration is associated with poorer cognitive function, raised core body temperature, increased glycogen utilisation, and increased sensation of fatigue, which may all lead to a negative effect on golf performance [5, 26]. It was therefore of no surprise that hydration and the golfer was one of the more researched areas of performance nutrition for golf. For example, the hydration status of 15 elite collegiate golfers (mean HCP + 1.1) was monitored using their urine specific gravity (USG) status immediately pre and post an 18-hole round [27]. For context a USG > 1.020 was considered dehydrated [28]. The mean number of shots taken were significantly higher (*p* = 0.049) in the players who began the round dehydrated (79.5 ± 2.1 strokes) when compared with the euhydrated group (75.7 ± 3.9 strokes). It was noted that all the dehydrated players (*n* = 6) failed to consume sufficient fluids throughout the 18 holes to reach a euhydrated state indicating an immediate target for golfer education. In support of this suggestion, the authors asked the golfers to complete the fluid section of a nutrition knowledge questionnaire, and it was reported that the golfers who started the round dehydrated displayed less knowledge on hydration compared with players in the euhydrated state. In a similar study using seven amateur golfers (HCP 3.0 ± 1.2), it was reported that cognitive tasks, including the golfers’ overall distance and accuracy judgements were impaired when even mildly dehydrated [5]. The dehydrated athletes experienced a body weight (BW) loss of just 1.1 kg, supporting previous suggestions that even modest levels of dehydration may be associated with performance decrements [26], in particular cognitive function [29].

There was limited research identified examining the composition of fluids provided during golfing performance. Thompsett et al. [18] investigated the effect of a zero-calorie drink (330 mL Gatorade Zero Sugar Orange Thirst Quencher) with or without 30 g carbohydrates or 15 g carbohydrates plus 15 g protein on fatigue levels and performance across 9 holes with feedings administered at holes 1, 4 and 7. In all conditions, the amateur golfers commenced the round somewhat dehydrated (USG of 1.021–1.023). The authors reported no effects of the drinks on golf performance or alertness but did suggest decreased levels of self-reported fatigue in the two carbohydrate conditions compared with the zero-calorie condition. This suggests that providing some nutrition with the fluid is better than fluid alone. However, it was not assessed if fluid alone was better than no fluid at all. It should also be noted that sweat rate during play was low (0.17–0.22 L/h) and therefore these data may not be applicable to golf in warmer climates where sweat rates may be significantly higher. Research on specific oral rehydration solutions and the specific composition of the drinks in golf is lacking, and it is therefore not possible to make definitive recommendations as to the ideal drinks to consume during competition. Future research should explore the composition of the drinks examining if electrolytes and/or carbohydrate solutions would be beneficial for golfing performance, and explore this in differing environmental conditions.

#### Energy Requirements of Golf

Studies on the energy expenditure of golf were the second-most prevalent topic of research reviewed with the prevailing view that golf provides moderate intensity exercise, while this varies within the round and between subjects and conditions (Table 2). However, it was apparent that due to a wide array of methodologies employed, combined with a wide range of playing standards, the range of reported energy expenditures was vast, ranging from 663 kcal to 1,954 kcal per round equating 3.2–11.8 kcal/min [30, 31]. Whilst the total amount of energy expended can depend on numerous factors including the individual, the method of transporting the clubs, climatic conditions, and course terrain, it appears the major discrepancy was related to the equipment employed to collect the energy expenditure data, which therefore must be taken into consideration when reviewing the data. For context, 11.8 kcal/min is greater than typically reported when running a marathon [32], which seems excessive for a sport whose primary activity is walking. Moreover, not all studies differentiated between the total energy expenditure of golf (total energy expended during the round) and activity energy expenditure of golf (total energy expended minus resting metabolic rate), which also may contribute to the variability reported.

**Table 2:** Energy expenditure (EE)/activity energy expenditure (AEE) over the course of 9 and 18 holes for various golfing abilities

Reference	Methodology	Total mean EE/AEE (kcal)	Mean EE/min (kcal/min)	Cohort
[35]	WHOOP Strap 2.0 worn, four rounds of 18 holes	1,555.8 ± 157.8 (AEE)	5.4 ± 0.4 kcal/min	20 challenge tour male golfers
[31]	ActiHeart^®^ Monitor. Three rounds (bag carrying (BC), electric trolley (ET) or push trolley (MT)) over three rounds of 18 holes	BC: 688 ± 213 MT: 756 ± 210 ET: 663 ± 218 (AEE)	BC: 3.4 kcal/min MT: 3.6 kcal/min ET: 3.2 kcal/min	16 golfers, HCP under 5 (14 males, 2 females)
[68]	Portable metabolic system worn using a motor cart (MC), pushcart (MT) and electric trolley (ET) over three rounds of 9 holes	No time noted (EE)	MC: 3.52 kcal/min MT: 4.8 kcal/min ET: 3.93 kcal/min	6 males, 4 females. Minimum age of 45
[34]	BioHarness 3 Zephyr-wireless Bluetooth wearable monitor and 3- dimensional accelerometer (Zephyr HxM, B. T. Heart Rate Monitor) worn over 18 holes	1,609.08 ± 527.9 kcal (EE)	6.58 kcal/min	25 participants (females, *n* = 11, males, *n* = 14) Mean HCP: 5.9 ± 2.9
[37] Thesis	Garmin VivoactiveHR™ worn for two rounds of 18 holes	1,642.3 ± 443.0 kcal (female) 1583.1 ± 145.8 kcal (male) (EE)	5.82 kcal/min (female) 5.95 kcal/min (male)	8 NCAA collegiate players (4 males/4 females)
[69] Conference proceedings	Actigraph accelerometer, a polar heart rate monitor, and Polar GPS worn carrying the bag [70] or riding a cart (PC) over two rounds of 9 holes	BC: 450.6 kcal PC: 265.6 kcal (EE)	No time noted	2 groups of 5 male subjects (mean age: 20.9 years old)
[71]	Global Positioning System device (FRWD W400 series) to the upper right arm and a pulsometer (FRWD W400 series) worn on the chest over four rounds of 18 holes	Player A: 1485.8 ± 72.3 kcal Player B: 1621.8 ± 80.5 kcal (EE)	Player A: 5.7 ± 0.03 kcal/min Player B: 6.19 ± 0.1 kcal/min	2 17-year-old male participants. HCP indexes: Player A: 10.1 Player B: 11.2
[41]	FRWD W400 series (Finland) device worn across 18 holes	1,084.1 ± 134.37 kcal (EE)	4.82 ± 0.53 kcal/min	10 male golfers (age: 16.7 ± 1.95 years) Mean HCP Index: 8.14 ± 3.52
[72]	SUUNTO T6c HR monitor worn over 9 or 18 holes	18-hole men: 926 ± 292 kcal 18-hole women: 556 ± 180 kcal 9-hole men: 520 ± 133 kcal 9-hole women: 273 ± 66 kcal (EE)	4.32 kcal/min 2.64 kcal/min 4.16 kcal/min 2.23 kcal/min	42 males, 24 females Average HCP at Hilly courses: 26.4 ± 12.5 (mixed) Flat courses: 20.4 ± 9.0 (mixed) Mean HCP: 10.9
[73] Thesis	Suunto HR monitor (Suunto t6c, Vantaa, Finland) and t6c watch was attached to the right wrist) over 18 holes whilst carrying their bags	1,202.8 ± 465.2 kcal (EE)	4.23 kcal/min	13 male subjects (20–58 years old). HCP range: 0–25
[74]	Portable metabolic system and global positioning system receiver worn across 9 holes	Gross golfing EE: 511.6 ± 115.5 Net golfing EE: 310.3 ± 83.9	4.19 kcal/min 2.54 kcal/min	18 males, 65–80 years of age
[30]	Portable telemetric metabolic system (K4b2, Cosmed USA) integrated with a heart rate monitor (S210, Polar USA) worn across 3 rounds of 18 holes, carrying their bag [70], using a caddy (C) or cart (B)	BC: 1954 kcal C: 1527 kcal B: 1303 kcal (EE)	11.3 kcal/min 8.84 kcal/min 6.44 kcal/min	1 43-year-old male subject

*B* cart, *BC* bag carry, *C* caddy, *EE* energy expenditure, *ET* electric trolley, *HCP* handicap, *HR* heart rate, *kcal* kilocalorie, *min* minute, *MT* push trolley, *PC* cart, *USA* United States of America

Kasper et al. [31] used Actiheart^®^ Monitors to measure the activity energy expenditure of golf over 18 holes in high-level golfers (HCP: 1.5 ± 2.4). The Actiheart^®^ Monitor has been validated against doubly labelled water (DLW) in free-living conditions [33] and may therefore provide a more reliable assessment of the activity energy expenditure of golf. Using Actiheart^®^ Monitors, some of the lowest energy expenditure values were reported (3.2 kcal/min, approximately 768 kcal/round) suggesting that the major contributor to the activity energy expenditure of golf is the walking between shots. Despite using validated equipment, the study was not without its limitations, which consisted of a higher percentage of males than females and the total walking distance covered during the rounds not being measured. This is important as it could be argued that higher standard golfers cover less distance than lower standard players as there may have been less searching for golf balls resulting in a more direct route being walked.

There have been suggestions that the energetic cost of golf differs in male versus female amateur golfers [34]. Using a BioHarness 3 Zephry-wireless monitor, Ilhan Odabas and Gercek reported that the female golfers had significantly higher energy expenditure (1823 ± 304 kcal; *n* = 11) than their male counterparts (1440 ± 611 kcal; *n* = 14), although both groups reported similar ratings of perceived exertion. The authors suggested that the difference in energy expenditure between the female participants and their male counterparts could be explained by the higher physiologic load, physiologic intensity, higher training load, and training intensity experienced by the females. The energetic costs were almost double that reported by Kasper et al. [31]. Future studies should further address this hypothesis in male and female golfers using the most appropriate techniques including DLW for the assessment of total daily energy expenditure, alongside ActiHeart^®^ Monitors to determine the energy expenditure of the individual components of golf play and practice (e.g., driving range, chipping, putting) and the playing round itself.

In terms of professional golf, the activity energy expenditure of 20 professional male golfers was measured over the course of four tournaments on The Challenge Tour and The Alps Tour using commercially available technology (WHOOP Strap 2.0) [35]. The Whoop Strap uses light-emitting diodes and photoplethysmography (PPG) to gather metrics such as heart rate and estimated basal metabolic rate (BMR) to assess activity energy expenditure [36]. Mean activity energy expenditure was 1555 ± 158 kcal per round, which also seems at the higher end for a low- to moderate-intensity sport [4]. It has been previously suggested [37] that using wearable technology to measure heart rate and then using this to estimate activity energy expenditure over 18 holes of golf overestimates energy expenditure (1609 kcal) given that heart rate may be affected by sensor movement, environment, and fitness [38, 39]. This may somewhat account for the high energy expenditures reported in golf when using heart rate technology. With such variability in the range of energy expenditure reported in the published golf literature, it is now essential to determine an accurate benchmark of the energy expenditure of golf play to ensure that golfers are fuelling themselves adequately for both their on-course performance and long-term health [40].

The method of transportation of clubs may also affect the energy expenditure of the round, especially when riding a golf cart. Research investigated the effects of one amateur subject riding a golf cart (1303 kcal) in comparison with using a caddy (1527 kcal) and carrying their own bag (1954 kcal) over 18 holes [30]. However, data may not be accurate as this was a case study on one amateur golfer and therefore requires replicating with a larger cohort of male and female golfers.

#### Nutrition and Cognitive Performance

Success in golf requires fine movement patterns and motor skills that require immense concentration. Due to the typical time taken to play a round of golf, there are many opportunities for a momentary lapse in concentration to occur and impact upon golf performance [41]. For example, cognitive anxiety, which is linked to negative self-talk, was shown to correlate with golf putting performance [42]. Participants reported that rational self-talk was more helpful for their putting performance when compared with irrational self-talk (mean difference = 16.49, SD = 28.38, *p* < 0.01). Furthermore, rational self-talk improved putting scores by 66% from baseline, in comparison with a 33% improvement when utilising irrational self-talk. Moreover, there have been suggestions of a link between elevated plasma cortisol concentrations and attentional bias towards negative self-talk, although there was no observed decrement in performance [43]. To our knowledge no study has assessed if nutrition can influence cortisol concentrations during a round of golf, which could have an impact on concentration and performance. However, the effects of 6 weeks of supplementation with 200 mg oral phosphatidylserine supplementation on perceived stress was investigated [44]. The authors reported a trend, albeit not statistically significant, for phosphatidylserine to reduce stress alongside a significant increase in ball flight accuracy. It should be stressed, however, that these data were collected on 20 mid-high HCP golfers and therefore should be translated to other standards of golfers with caution [45]. Future research should assess if nutritional strategies can reduce cognitive anxiety and improve aspects of golf mental performance. Such research should not only assess if nutritional supplements may help but also assess the impacts of maintaining euhydration and optimised on-course fuelling strategies. There are suggestions that hydration may impact cognitive performance (through misjudgement of distance) when dehydrated [5], and this should be further explored.

### Dietary Supplements and Golf Performance

Whilst it is important that athletes, including golfers, should adhere to a food-first strategy, there are a number of situations where performance supplements may be considered using a food-first but not always food-only approach [46]. It is strongly advised that golfers, especially those competing at a high level (e.g., national squads, professional), only consider use of supplements with caution due to the risk of potential anti-doping rule violations [52]. To mitigate these risks, it is advised players consult with qualified professionals to ensure that comprehensive risk-mitigation strategies are employed including batch testing by a reputable third-party organisation. This scoping review identified a small number of studies on performance supplements for golf, although the research was somewhat limited when compared with supplement research in other sports.

#### Caffeine

Caffeine is one of the most widely studied ergogenic aids, with research suggesting that it can support cognitive performance [47] and enhance endurance exercise [48] and improve team-sport performance [49]. An effective dose of caffeine ranges from 3 to 6 mg/kg BM [47], with a typical 250 mg oral dose of caffeine achieving peak plasma concentrations within 45–60 min post consumption [48]. While multiple studies have explored the effects of caffeine on endurance capacity during extended exercise, to date there has been limited research on golf performance. As a full round of golf typically lasts 4–6 h, and can also be played over consecutive days, caffeine consumed before or during a round may help to maintain plasma caffeine concentrations and alleviate symptoms of fatigue. As some tee times occur later in the day, golfers need to be cautious when using caffeine, as this may negatively impact sleep quality, leading to impaired performance in the following days.

Mumford et al. [50] examined the effect of a caffeine-containing supplement on golf-specific performance and fatigue throughout a 36-hole competitive tournament. In a double-blind, placebo-controlled study, 12 male golfers (HCP 3–10) consumed either a 155 mg caffeine supplement or a placebo 25–35 min before and after each 9 holes over two consecutive days. Caffeine intake led to significantly better total scores (76.9 ± 8.1 vs. 79.4 ± 9.1, *p* = 0.039), a greater number of ‘greens in regulation’ (8.6 ± 3.3 vs. 6.9 ± 4.6, *p* = 0.035), and increased drive distance (239.9 ± 33.8 vs. 233.2 ± 32.4 yards, *p* = 0.047). The caffeine group also reported greater feelings of energy mid round (*p* = 0.025).

Stevenson et al. examined the effects of a combined (commercially available) caffeine and carbohydrate sport drink on golf putting performance in 20 male amateur golfers (HCP 15 ± 4) during a laboratory-based simulated golf round [6]. The authors reported that the caffeine-containing carbohydrate drink (1.6 mg/kg BM caffeine with 0.64 g/kg carbohydrate) consumed before and during a round of golf led to improved putting performance and increased alertness. Across all 18 holes, the caffeine-containing carbohydrate drink resulted in a significantly higher number of successful putts and significantly lower number of putts falling short of the hole. There was also a main effect of drink on self-rated scores for alertness and relaxation. It should be stressed, however, that the study was not able to differentiate between the effects of caffeine and carbohydrate on golf performance. Not all studies have reported improvements in golf performance following caffeine supplementation. Bristow [51] reported that there was no significant difference between 11 male golfers (HCP: 4.8 ± 3.7) in any performance variables over the course of an 18-hole round between caffeine (3 mg/kg) and a placebo. However, when ten drives were hit on a golf simulator, the caffeine group demonstrated significant improvements in ball speed and total distance. Taken together, there are suggestions that caffeine may enhance aspects of golf performance when taken in the 1.5–3 mg/kg dose range, although more research is needed to explore this as well as assess if doses can be detrimental to golf performance, particularly in caffeine-naïve players. The effects of subsequent days’ performance given potential impact on sleep also needs to be considered.

#### Creatine

Although in theory creatine monohydrate supplementation could be useful for golfing physical and mental performance [52, 53], golf-specific research on creatine supplementation is limited. The only study identified in the present scoping review used a multi-ingredient commercially available supplement containing 5 g creatine along with 50 mg coffee extract, calcium fructoborate and vitamin D, and compared this to an isocaloric placebo control. In male golfers (5–15 HCP), following 30 days of supplementation the mean driving distance in the experimental group increased (from 270 ± 19 to 284 ± 23 yards) alongside improvements in BM and peak power and velocity during bench press throws [54]. Although the product was a multi-ingredient drink containing caffeine, the actual caffeine dose was low and as such it is likely that the improvement was a result of the creatine, although this suggestion remains speculative. Given the potential for creatine to aid golfing performance, future research should explore this hypothesis investigating the effects of creatine supplementation on both the physical and cognitive aspects of golfing performance.

### Body Composition, Anthropometric Profiles and Golf Performance

Several studies identified in the present scoping review investigated the body composition of golfers across various skill levels [16, 21]. Golfers tended to have higher body fat compared with athletes in more aerobically demanding sports, such as football [55, 56] and rugby [57]. However, within the golfing population, lower HCP players (who tend to perform better) were reported to have lower body fat percentages alongside greater lean BM compared with higher HCP players [55, 58] suggesting that body composition may be important to golfing performance. Kawashima et al. [55] compared the body composition of professional and amateur Japanese male golfers, showing that professional players had significantly lower body fat percentages (12.8 vs. 19.8%) and higher lean BM than amateur players. It must be stressed that body fat was measured using skinfold callipers, which has limitations when translating from a skinfold thickness to a body fat percentage [59]. Previous work [58] suggested that college female golfers had an average body fat percentage of 28 ± 6%, whilst male golfers had an average body fat percentage of 19 ± 7%, although this again involved skinfold thickness and prediction equations.

More recently, the fat-free mass index (a height-adjusted measure of fat-free mass) of collegiate golfers was investigated [60]; this index is calculated by dividing an individual’s fat-free mass by their height squared [61]. The mean fat-free mass index for male collegiate golfers was 21 ± 1.5 kg·m^2^, which was lower than that of collegiate rugby and football players. A higher fat-free mass index indicates a higher relative muscularity, which may be beneficial for golfers as higher muscle mass is associated with increased strength, power and driving distance [60, 62]. There was insufficient literature to conclude on an ideal or preferred body composition for golf, and therefore additional research is needed to help with talent identification and to direct the off-course training requirements of golfers. Future studies should attempt to assess this, ideally using a combination of techniques including dual-energy X-ray absorptiometry (DXA) scan technology in a wide range of male and female golfers.

### Travel and Golf

Golfers of all levels frequently travel domestically and internationally for tournaments and training, which can lead to additional unwanted stress on the body, travel fatigue, jet lag, dehydration and gastrointestinal (GI) issues that may negatively impact health and performance [63]. Golfers can utilise individualised nutrition strategies to manage travel fatigue, jet lag and illness when travelling for competitions and training. No studies were identified that looked specifically at the effects of nutrition and travel for golfers, but literature focusing on various other sports and strategies for travel has been explored.

Despite there being no studies specifically focusing on the effects of probiotics and golf, there is available evidence that suggests positive associations with probiotics decreasing upper respiratory tract infections (URTIs) and GI distress, especially when travelling [64]. With the significant travel and competition demands in competitive golf, probiotic supplementation and/or probiotic-containing foods could decrease the risk of illness, although golf-specific studies are now required to explore this. Moreover, despite a recent meta-analysis demonstrating that zinc acetate lozenges may decrease the duration and symptoms of URTIs [65], again there are no golf-specific studies examining this. Given the lack of specific research in this area, the suggested nutrition strategies to reduce the stress burden for golfers documented in a golf text book [66] and a narrative review [63] were forced to utilise best practice guidance from other sports.

General recommendations to help reduce travel fatigue and jet lag suggest that golfers should maintain hydration on the plane, with the possible use of electrolytes, adjust meal timing to the destination, and consider the strategic use of caffeine [66] whilst preventing any nutrient deficiencies such as low vitamin D [63]. Moreover, nutrition suggestions that help to overcome jet-lag symptoms may also aid with day-to-day performance situations given the highly variable tee times players can experience (e.g., waking up at 5 a.m. for an early tee time one day, and then having an afternoon tee time the following day). These suggestions now must be explored in golf-specific research to see if there are any definitive suggestions that can be applied in the sport as most literature has been conducted on sedentary individuals or the general population and not elite athletes.

### Future Research Priorities

This scoping review has identified areas of research that should be focussed on in the future and which are summarised in Table 3. Immediate areas to address include clarification of the confusion with regards to the energetic demands of the sport using techniques such as DLW [67], followed by better understanding of the dietary carbohydrate, protein and fat requirements of elite and recreational players. Despite body composition being the most frequently researched topic in golf, we also suggest that future research should aim to clarify the body composition and anthropometric characteristics of male and female players using improved methodologies such as DXA. To our knowledge, most golf research has been conducted using males, with limited high-quality research on female golfers, and therefore future research should focus on studying larger cohorts of female players of all abilities using accurate methodologies. Given the lack of literature regarding the efficacy of dietary supplements (including caffeine, creatine, electrolytes and supplements reported to enhance cognitive function), there is a need for studies to thoroughly examine such supplements within the context of golf and investigate if any of these nutritional strategies may provide benefits to the golfer. It has been suggested that recovery is a key consideration for the modern golfer given their intense playing schedule, prolonged season and high travel demands [66]. Despite the importance of recovery, it was surprising to note there was no research on nutritional strategies to assist recovery from intense playing schedules, and future research should attempt to address this gap in the literature. Finally, research regarding the nutrition knowledge of golfers would be informative to help understand the barriers to implementing targeted sport nutrition support within golf.

**Table 3:** Research priorities relating to golf and nutrition

Research priority relating to golf	Requirements	Why required
Nutrition and hydration	Research involving specifically golf for nutrition/ hydration recommendations is scarce and recommendations currently obtained from general guidelines and other sports	Weight of evidence low
Macronutrient distribution	Literature exploring the utilisation of carbohydrates and fat as a fuel source when competing. Gaining an idea of an ideal carbohydrate intake for golfers of all abilities	Knowledge gap
Energy expenditure	More validated research needed exploring an accurate measurement of EE especially for females and professional golfers. Areas such as methods of transporting the clubs/ different climates/ different courses should be explored	Weight of evidence low
Body composition	More research exploring the relationship between the ideal body composition and golf and how nutrition strategies may help	Weight of evidence low
Cognitive performance	Research to explore the relationship between nutrition and cognitive function/ alertness in relation to golf	Weight of evidence low
Supplements	Research analysing potential supplement use that may enhance golf performance is needed. For example, investigating the potential benefits of electrolytes considering the several hot climates tournaments are played in	Weight of evidence low
Specific populations	Research addressing associations between golf and nutrition/ hydration in (1) females and (2) professional athletes. This may highlight specific nutrition differences/energy requirements depending on players' gender/ability	Weight of evidence low
Travel	An area under researched, especially in the elite/professional cohort. Research may address common struggles/trends about nutrition on tour (e.g., accessible food availability, variation, common travel illnesses, potential benefits of zinc acetate)	Knowledge gap

*EE* energy expenditure

### Limitations

This scoping review has limitations. Firstly, our search was limited to articles written or translated into the English language, therefore other relevant articles in various languages may have been missed. This may also account for countries where English is the first language (USA, UK and Australia) featuring prominently in the included studies. Secondly, we identified articles from the last 20 years to try and provide the most up-to-date and accurate research, although we do acknowledge that older literature does have merit and may have proved relevant. In keeping with scoping review methodology, the studies included were not formally assessed for the risk of bias. However, scoping reviews are broad in nature and aim to provide a more extensive audit of the literature in comparison with a systematic review [13]. Nevertheless, this review does provide an extensive summary of nutrition in relation to golf.

## Conclusions

This scoping review identified 82 articles relevant to the specific question, “Where does the current research lie with regards to nutrition, hydration, energy requirements and its effects on golf and performance?” Despite focussing the scoping review on nutrition, the most prevalent literature focussed upon body composition and the anthropometric profiles of golfers. We suggest that there is currently a lack of golf-specific nutrition, hydration and energy guidelines for golfers of all abilities, and where there was nutrition literature, the vast majority has been conducted using small cohorts of recreational male golfers often using less than ideal research methodologies. We therefore suggest that future research within the nutrition and golf domain must now use more valid methodologies using larger cohorts of male and female golfers with some of this conducted on the elite professional player.

### Key Findings/Implications

This research highlights that the consumption of carbohydrates during a round may be of benefit to performance. Dehydration may negatively impact upon performance including reduced driving distance and accuracy judgement. Current energy expenditure data reported has a broad range of 2.23–11.3 kcal/min. This appears to be too varied to be accurate and therefore must be clarified in future research. Creatine and caffeine may be a beneficial addition for the golfer to improve performance, whilst zinc acetate lozenges and probiotics could help prevent illness whilst travelling. Future areas of interest include golf-specific nutrition and hydration recommendations, macronutrient requirements for golf, clarification of the energy expenditure, assessment of the preferred body composition for the elite golfer, how to improve cognitive performance, golf-specific sports supplements, and strategies to assist with the extensive travel.

## Supplementary Information

Below is the link to the electronic supplementary material.

Supplementary file1 (PDF 347 KB)
