# Supplementary material for: Nutrition and Golf Performance: A Systematic Scoping Review
Source: Sports Med. 2024 Sep 30;54(12):3081–95. doi: 10.1007/s40279-024-02095-0 (PMC11608286; doi:10.1007/s40279-024-02095-0)
Supplement: Supplementary file 1 — Supplementary file1 (PDF 347 KB) [file 40279_2024_2095_MOESM1_ESM.pdf]

Electronic Supplementary Material- Key Words and Search Strings

Nutrition and Golf Performance: A Systematic Scoping Review

O'Donnell, A[1,2,3], Murray, AD[1,2,3, 4], Nguyen, A[1], Salmon[1], T, Taylor[1], S, Morton, JP[1] and Close, GL[1,3,4]

1. Research Institute for Sport and Exercise Sciences
   Liverpool John Moores University
   Liverpool, UK

2. Ladies European Tour Performance Institute
   Denham
   UK

3. Medical and Scientific Department
   The R&A
   St Andrews,
   UK

4. PGA European Tour Health and Performance Institute,
   Virginia Water,
   UK

Sports Medicine

Address for correspondence:
Professor Graeme L. Close
Research Institute for Sport and Exercise Sciences
Liverpool John Moores University
Liverpool, UK
L3 3AF
g.l.close@ljmu.ac.uk
0151 904 6266

Running Title: Nutrition and Golf

Search Strategy from ProQuest

21 December 2023 12:45

SEARCH STRATEGY

Set No.	Searched for	Databases	Results
S4	[S1] AND [S3]	ProQuest Central<br>These databases are searched for part of your query.	28
S5	ABSTRACT,TITLE(("energy expenditure" OR "energy expenditures") OR "activity energy expenditure*" OR "energy intake" OR "caloric cost" OR "energy cost" OR "metabolic cost" OR calorie*) AND ABSTRACT,TITLE(("carry bag" OR "carry bags") OR ("electric trolley" OR "electric trolleybus" OR "electric trolleybuses" OR "electric trolleys") OR "push trolley*" OR ("golf cart" OR "golf carts") OR ("golf buggy") OR "single strap bag*" OR "double strap bag*")	ProQuest Central<br>These databases are searched for part of your query.	3
S6	(ABSTRACT,TITLE(("nine hole" OR "nine holes") OR "9 hole*" OR ("eighteen hole" OR "eighteen holes") OR "18 hole*" OR "simulated play" OR "simulated round*" OR "driving range") NOT stype.exact("Newspapers" OR "Wire Feeds" OR "Trade Journals" OR "Blogs, Podcasts, & Websites" OR "Magazines" OR "Audio & Video Works")) AND ABSTRACT,TITLE(Caffeine* OR creatine OR supplement*)	ProQuest Central<br>These databases are searched for part of your query.	28
S13	(ABSTRACT,TITLE(Caffeine* OR creatine OR supplement*) NOT stype.exact("Newspapers" OR "Wire Feeds" OR "Trade Journals" OR "Blogs, Podcasts, & Websites" OR "Magazines" OR "Audio & Video Works")) NOT stype.exact("Reports")	ProQuest Central	327386
S14	ABSTRACT,TITLE(golf*) NOT	ProQuest Central	11401

	stype.exact("Newspapers" OR "Wire Feeds" OR "Trade Journals" OR "Blogs, Podcasts, & Websites" OR "Magazines" OR "Audio & Video Works")	These databases are searched for part of your query.	
S15	ABSTRACT,TITLE(golf*) NOT stype.exact("Newspapers" OR "Wire Feeds" OR "Trade Journals" OR "Blogs, Podcasts, & Websites" OR "Magazines" OR "Audio & Video Works") NOT stype.exact("Reports")	ProQuest Central<br>These databases are searched for part of your query.	9585
S16	[S13] AND [S15]	ProQuest Central<br>These databases are searched for part of your query.	52
S17	ABSTRACT,TITLE(carbohydrate* OR protein* OR fat* OR macronutrient* OR micronutrient* OR vitamin*)	ProQuest Central	4612709
S18	ABSTRACT,TITLE(carbohydrate* OR protein* OR fat* OR macronutrient* OR micronutrient* OR vitamin*) NOT stype.exact("Newspapers" OR "Wire Feeds" OR "Trade Journals" OR "Blogs, Podcasts, & Websites" OR "Magazines" OR "Reports" OR "Standards & Practice Guidelines")	ProQuest Central	2013034
S19	ABSTRACT,TITLE(golf*) NOT stype.exact("Newspapers" OR "Wire Feeds" OR "Trade Journals" OR "Blogs, Podcasts, & Websites" OR "Magazines" OR "Reports" OR "Standards & Practice Guidelines")	ProQuest Central	9628
S20	[S18] AND [S19]	ProQuest Central<br>These databases are searched for part of your query.	236
S21	[S6] AND [S18]	ProQuest Central<br>These databases are searched for part of your query.	6
S22	ABSTRACT,TITLE(Performance* OR "Golf* Performance*") NOT stype.exact("Newspapers" OR "Wire Feeds" OR "Trade	ProQuest Central	2650589

	Journals" OR "Blogs, Podcasts, & Websites" OR "Magazines" OR "Reports" OR "Standards & Practice Guidelines")		
S23	[S16] AND [S22]	ProQuest Central<br>These databases are searched for part of your query.	14
S24	[S20] AND [S22]	ProQuest Central<br>These databases are searched for part of your query.	29
S25	ABSTRACT,TITLE(("energy expenditure" OR "energy expenditures") OR "activity energy expenditure*" OR "energy intake" OR "caloric cost" OR "energy cost" OR "metabolic cost" OR calorie*)	ProQuest Central	104918
S26	ABSTRACT,TITLE(("energy expenditure" OR "energy expenditures") OR "activity energy expenditure*" OR "energy intake" OR "caloric cost" OR "energy cost" OR "metabolic cost" OR calorie*) NOT stype.exact("Newspapers" OR "Trade Journals" OR "Wire Feeds" OR "Blogs, Podcasts, & Websites" OR "Magazines" OR "Reports" OR "Standards & Practice Guidelines" OR "Audio & Video Works")	ProQuest Central	53795
S27	[S19] AND [S26]	ProQuest Central<br>These databases are searched for part of your query.	22
S28	[S22] AND [S27]	ProQuest Central<br>These databases are searched for part of your query.	5
S29	ABSTRACT,TITLE("Challenge Tour" OR "DP World Tour" OR "European Tour" OR "PGA" OR "PGA TOUR" OR NCAA OR "Ladies European Tour" OR "LET" OR "Ladies Professional Golf Association" OR "LPGA") NOT stype.exact("Newspapers" OR "Trade Journals" OR "Wire Feeds" OR "Blogs, Podcasts, & Websites" OR "Magazines" OR "Reports" OR "Standards & Practice Guidelines" OR	ProQuest Central	136804

	"Audio & Video Works")		
S30	ABSTRACT,TITLE(nutrition* OR diet* OR food OR "sport* nutrition*") NOT stype.exact("Newspapers" OR "Trade Journals" OR "Wire Feeds" OR "Blogs, Podcasts, & Websites" OR "Magazines" OR "Reports" OR "Standards & Practice Guidelines" OR "Audio & Video Works")	ProQuest Central	941613
S31	[S22] AND [S29] AND [S30]	ProQuest Central<br>These databases are searched for part of your query.	115
S32	[S19] AND [S31]	ProQuest Central<br>These databases are searched for part of your query.	2
S33	ABSTRACT,TITLE("putting accuracy*" OR "putting performance" OR putting OR "clubhead speed*" OR "swing speed*" OR "clubhead velocity" OR "drive distance" OR "driving accuracy" OR "shot precision*") NOT stype.exact("Newspapers" OR "Trade Journals" OR "Wire Feeds" OR "Blogs, Podcasts, & Websites" OR "Magazines" OR "Reports" OR "Standards & Practice Guidelines" OR "Audio & Video Works")	ProQuest Central	40395
S34	[S30] AND [S33]	ProQuest Central<br>These databases are searched for part of your query.	1410
S35	[S19] AND [S34]	ProQuest Central<br>These databases are searched for part of your query.	6
S36	[S20] AND [S33]	ProQuest Central<br>These databases are searched for part of your query.	10
S37	[S13] AND [S19] AND [S33]	ProQuest Central<br>These databases are searched for part of your query.	2
S38	ABSTRACT,TITLE("Amateur*" OR "Professional*" OR ("elite amateur") OR ("semi professional" OR "semi professionalism" OR "semi professionally" OR "semi professionals") OR recreational) NOT stype.exact("Newspapers" OR "Trade Journals" OR "Wire Feeds" OR "Blogs, Podcasts,	ProQuest Central	574215

	& Websites" OR "Magazines" OR "Reports" OR "Standards & Practice Guidelines" OR "Audio & Video Works")		
S39	[S30] AND [S33] AND [S38]	ProQuest Central<br>These databases are searched for part of your query.	60
S40	[S19] AND [S39]	ProQuest Central<br>These databases are searched for part of your query.	0
S41	ABSTRACT,TITLE(glucose* OR "energy gel*" OR gel* OR "isotonic gel*") NOT stype.exact("Newspapers" OR "Trade Journals" OR "Wire Feeds" OR "Blogs, Podcasts, & Websites" OR "Magazines" OR "Reports" OR "Standards & Practice Guidelines" OR "Audio & Video Works")	ProQuest Central	408660
S42	[S19] AND [S41]	ProQuest Central<br>These databases are searched for part of your query.	39
S43	[S33] AND [S42]	ProQuest Central<br>These databases are searched for part of your query.	3
S44	[S19] AND [S22] AND [S41]	ProQuest Central<br>These databases are searched for part of your query.	5
S45	[S29] AND [S41]	ProQuest Central<br>These databases are searched for part of your query.	821
S46	[S19] AND [S29] AND [S41]	ProQuest Central<br>These databases are searched for part of your query.	0
S47	[S6] AND [S41]	ProQuest Central<br>These databases are searched for part of your query.	1
S48	[S27] AND [S41]	ProQuest Central<br>These databases are searched for part of your query.	2
S49	ABSTRACT,TITLE(fluid* OR electrolyte*) NOT stype.exact("Newspapers" OR "Trade Journals" OR "Wire Feeds" OR "Blogs, Podcasts, & Websites" OR "Magazines" OR "Reports" OR "Standards & Practice Guidelines" OR "Audio & Video Works")	ProQuest Central	514079
S50	[S19] AND [S49]	ProQuest Central<br>These databases are searched for part of your query.	50
S51	[S33] AND [S49]	ProQuest Central<br>These databases are searched for part of your query.	301
S52	[S33] AND [S50]	ProQuest Central<br>These databases are searched for part of your query.	2
S53	[S19] AND [S29] AND [S49]	ProQuest Central<br>These databases are searched for part of your query.	0

S54	[S6] AND [S49]	ProQuest Central<br>These databases are searched for part of your query.	0
S55	ABSTRACT,TITLE(hydration OR dehydration OR euhydration) NOT stype.exact("Newspapers" OR "Trade Journals" OR "Wire Feeds" OR "Blogs, Podcasts, & Websites" OR "Magazines" OR "Reports" OR "Standards & Practice Guidelines" OR "Audio & Video Works")	ProQuest Central	46870
S56	[S19] AND [S55]	ProQuest Central<br>These databases are searched for part of your query.	9
S57	[S29] AND [S55]	ProQuest Central<br>These databases are searched for part of your query.	86
S58	[S19] AND [S29] AND [S55]	ProQuest Central<br>These databases are searched for part of your query.	0
S59	[S6] AND [S55]	ProQuest Central<br>These databases are searched for part of your query.	0
S60	[S19] AND [S22] AND [S56]	ProQuest Central<br>These databases are searched for part of your query.	3
S61	[S6] AND [S55]	ProQuest Central<br>These databases are searched for part of your query.	0
S62	[S29] AND [S55]	ProQuest Central<br>These databases are searched for part of your query.	86
S63	[S19] AND [S62]	ProQuest Central<br>These databases are searched for part of your query.	0
S64	[S22] AND [S56]	ProQuest Central<br>These databases are searched for part of your query.	3
S65	[S33] AND [S56]	ProQuest Central<br>These databases are searched for part of your query.	1
S66	[S49] AND [S56]	ProQuest Central<br>These databases are searched for part of your query.	2
S67	ABSTRACT,TITLE(("carry bag" OR "carry bags") OR ("electric trolley" OR "electric trolleybus" OR "electric trolleybuses" OR "electric trolleys") OR "push trolley*" OR ("golf cart" OR "golf carts") OR ("golf buggy") OR "single strap bag*" OR "double strap bag*") NOT stype.exact("Newspapers" OR "Trade Journals" OR "Wire Feeds" OR "Blogs, Podcasts, & Websites" OR "Magazines" OR "Reports" OR "Standards	ProQuest Central	243

	& Practice Guidelines" OR "Audio & Video Works")		
S68	[S55] AND [S67]	ProQuest Central<br>These databases are searched for part of your query.	0
S69	[S41] AND [S67]	ProQuest Central<br>These databases are searched for part of your query.	1
S70	[S26] AND [S67]	ProQuest Central<br>These databases are searched for part of your query.	3
S71	ABSTRACT,TITLE("sweat rate*" OR sweat*) NOT stype.exact("Newspapers" OR "Trade Journals" OR "Wire Feeds" OR "Blogs, Podcasts, & Websites" OR "Magazines" OR "Reports" OR "Standards & Practice Guidelines" OR "Audio & Video Works")	ProQuest Central	14445
S72	[S19] AND [S71]	ProQuest Central<br>These databases are searched for part of your query.	9
S73	[S29] AND [S71]	ProQuest Central<br>These databases are searched for part of your query.	83
S74	[S19] AND [S29] AND [S71]	ProQuest Central<br>These databases are searched for part of your query.	0
S75	[S6] AND [S71]	ProQuest Central<br>These databases are searched for part of your query.	0
S76	[S67] AND [S71]	ProQuest Central<br>These databases are searched for part of your query.	0
S77	ABSTRACT,TITLE("cognitive function" OR "decision making" OR "motor function") NOT stype.exact("Newspapers" OR "Trade Journals" OR "Wire Feeds" OR "Blogs, Podcasts, & Websites" OR "Magazines" OR "Reports" OR "Standards & Practice Guidelines" OR "Audio & Video Works")	ProQuest Central	355570
S78	[S19] AND [S77]	ProQuest Central<br>These databases are searched for part of your query.	70
S79	[S30] AND [S78]	ProQuest Central<br>These databases are searched for part of your query.	2
S80	[S18] AND [S78]	ProQuest Central<br>These databases are searched for part of your query.	2
S81	[S55] AND [S78]	ProQuest Central<br>These databases are searched for part of your query.	1
S82	[S13] AND [S78]	ProQuest Central<br>These databases are searched for part of your query.	3
S83	ABSTRACT,TITLE("Heart Rate Monitor*" OR "Actiheart	ProQuest Central	2116

	Rate Monitor*" OR "Actiheart Monitor*" OR "Actiheart*") NOT stype.exact("Newspapers" OR "Trade Journals" OR "Wire Feeds" OR "Blogs, Podcasts, & Websites" OR "Magazines" OR "Reports" OR "Standards & Practice Guidelines" OR "Audio & Video Works")		
S84	[S19] AND [S83]	ProQuest Central<br>These databases are searched for part of your query.	4
S85	[S19] AND [S26] AND [S83]	ProQuest Central<br>These databases are searched for part of your query.	1
S86	[S67] AND [S83]	ProQuest Central<br>These databases are searched for part of your query.	0
S87	[S29] AND [S83]	ProQuest Central<br>These databases are searched for part of your query.	8
S88	[S22] AND [S84]	ProQuest Central<br>These databases are searched for part of your query.	1
S89	[S6] AND [S84]	ProQuest Central<br>These databases are searched for part of your query.	0
S90	[S26] AND [S67]	ProQuest Central<br>These databases are searched for part of your query.	3
S91	ABSTRACT,TITLE(("heart rate" OR "heart rates") OR ("heart beat" OR "heart beating" OR "heart beats")) NOT stype.exact("Newspapers" OR "Trade Journals" OR "Wire Feeds" OR "Blogs, Podcasts, & Websites" OR "Magazines" OR "Reports" OR "Standards & Practice Guidelines" OR "Audio & Video Works")	ProQuest Central	75318
S92	[S19] AND [S91]	ProQuest Central<br>These databases are searched for part of your query.	47
S93	[S26] AND [S92]	ProQuest Central<br>These databases are searched for part of your query.	6
S94	[S55] AND [S92]	ProQuest Central<br>These databases are searched for part of your query.	1
S95	[S30] AND [S92]	ProQuest Central<br>These databases are searched for part of your query.	2
S96	[S18] AND [S92]	ProQuest Central<br>These databases are searched for part of your query.	5
S97	[S13] AND [S92]	ProQuest Central<br>These databases are searched for part of your query.	2
S98	[S77] AND [S92]	ProQuest Central	2

		These databases are searched for part of your query.	
S99	[S67] AND [S92]	ProQuest Central<br>These databases are searched for part of your query.	3
S100	[S33] AND [S92]	ProQuest Central<br>These databases are searched for part of your query.	19
S101	[S13] AND [S100]	ProQuest Central<br>These databases are searched for part of your query.	0
S102	[S22] AND [S92]	ProQuest Central<br>These databases are searched for part of your query.	29
S103	ABSTRACT,TITLE(("body composition") OR "weight" OR "lean muscle mass" OR "fat free mass" OR "adipose tissue") NOT stype.exact("Newspapers" OR "Trade Journals" OR "Wire Feeds" OR "Blogs, Podcasts, & Websites" OR "Magazines" OR "Reports" OR "Standards & Practice Guidelines" OR "Audio & Video Works")	ProQuest Central	687607
S104	[S19] AND [S103]	ProQuest Central<br>These databases are searched for part of your query.	215
S105	[S29] AND [S104]	ProQuest Central<br>These databases are searched for part of your query.	6
S106	[S33] AND [S104]	ProQuest Central<br>These databases are searched for part of your query.	20
S107	ABSTRACT,TITLE(("dietary intake" OR "dietary intakes") OR "energy intake" OR "calorie intake") NOT stype.exact("Newspapers" OR "Trade Journals" OR "Wire Feeds" OR "Blogs, Podcasts, & Websites" OR "Magazines" OR "Reports" OR "Standards & Practice Guidelines" OR "Audio & Video Works")	ProQuest Central	34999
S108	[S19] AND [S107]	ProQuest Central<br>These databases are searched for part of your query.	3
S109	[S29] AND [S107]	ProQuest Central<br>These databases are searched for part of your query.	41
S110	[S19] AND [S109]	ProQuest Central<br>These databases are searched for part of your query.	0
S111	ABSTRACT,TITLE("resting metabolic rate*" OR "RMR" OR "basal metabolic rate*" OR "BMR") NOT stype.exact("Newspapers" OR	ProQuest Central	4634

	"Trade Journals" OR "Wire Feeds" OR "Blogs, Podcasts, & Websites" OR "Magazines" OR "Reports" OR "Standards & Practice Guidelines" OR "Audio & Video Works")		
S112	[S19] AND [S111]	ProQuest Central<br>These databases are searched for part of your query.	3
S113	[S29] AND [S111]	ProQuest Central<br>These databases are searched for part of your query.	13
S114	[S6] AND [S111]	ProQuest Central<br>These databases are searched for part of your query.	0
S115	[S18] AND [S112]	ProQuest Central<br>These databases are searched for part of your query.	1
S116	[S30] AND [S111] AND [S112]	ProQuest Central<br>These databases are searched for part of your query.	3
S117	ABSTRACT,TITLE("Physiological demand*" OR "metabolic demand*" OR physiologic*) NOT stype.exact("Newspapers" OR "Trade Journals" OR "Wire Feeds" OR "Blogs, Podcasts, & Websites" OR "Magazines" OR "Reports" OR "Standards & Practice Guidelines" OR "Audio & Video Works")	ProQuest Central	307748
S118	[S19] AND [S117]	ProQuest Central<br>These databases are searched for part of your query.	78
S119	[S29] AND [S117]	ProQuest Central<br>These databases are searched for part of your query.	474
S120	[S29] AND [S117] AND [S19]	ProQuest Central<br>These databases are searched for part of your query.	3
S121	[S67] AND [S118]	ProQuest Central<br>These databases are searched for part of your query.	3
S122	[S30] AND [S118]	ProQuest Central<br>These databases are searched for part of your query.	9
S123	[S55] AND [S118]	ProQuest Central<br>These databases are searched for part of your query.	1
S124	ABSTRACT,TITLE(physiology OR physiologic*) NOT stype.exact("Newspapers" OR "Trade Journals" OR "Wire Feeds" OR "Blogs, Podcasts, & Websites" OR "Magazines" OR "Reports" OR "Standards & Practice Guidelines" OR "Audio & Video Works")	ProQuest Central	367427
S125	[S19] AND [S124]	ProQuest Central	89

		These databases are searched for part of your query.	
S126	[S67] AND [S125]	ProQuest Central<br>These databases are searched for part of your query.	1
S127	[S29] AND [S124]	ProQuest Central<br>These databases are searched for part of your query.	582
S128	[S29] AND [S124] AND [S19]	ProQuest Central<br>These databases are searched for part of your query.	4
S129	[S38] AND [S125]	ProQuest Central<br>These databases are searched for part of your query.	16
S130	[S30] AND [S125]	ProQuest Central<br>These databases are searched for part of your query.	13
S131	ABSTRACT,TITLE("caloric* expenditure*") NOT stype.exact("Newspapers" OR "Trade Journals" OR "Wire Feeds" OR "Blogs, Podcasts, & Websites" OR "Magazines" OR "Reports" OR "Standards & Practice Guidelines" OR "Audio & Video Works")	ProQuest Central	269
S132	[S19] AND [S131]	ProQuest Central<br>These databases are searched for part of your query.	2
S133	[S67] AND [S132]	ProQuest Central<br>These databases are searched for part of your query.	0
S134	[S29] AND [S132]	ProQuest Central<br>These databases are searched for part of your query.	0
S135	ABSTRACT,TITLE("energy availability") NOT stype.exact("Newspapers" OR "Trade Journals" OR "Wire Feeds" OR "Blogs, Podcasts, & Websites" OR "Magazines" OR "Reports" OR "Standards & Practice Guidelines" OR "Audio & Video Works")	ProQuest Central	1801
S136	[S19] AND [S135]	ProQuest Central<br>These databases are searched for part of your query.	2
S137	[S29] AND [S135]	ProQuest Central<br>These databases are searched for part of your query.	8
S138	ABSTRACT,TITLE(("golf course" OR "golf courses") OR "golf terrain") NOT stype.exact("Newspapers" OR "Trade Journals" OR "Wire Feeds" OR "Blogs, Podcasts, & Websites" OR "Magazines" OR "Reports" OR "Standards & Practice Guidelines" OR "Audio & Video Works")	ProQuest Central	1673

S139	[S26] AND [S138]	ProQuest Central<br>These databases are searched for part of your query.	4
S140	[S30] AND [S138]	ProQuest Central<br>These databases are searched for part of your query.	58
S141	[S30] AND [S38] AND [S138]	ProQuest Central<br>These databases are searched for part of your query.	6

Database copyright © 2023 ProQuest LLC. All rights reserved.

Terms and Conditions Contact ProQuest

**Search Strings**
"golf*" (T) OR (A)
"Nine hole*" OR "9 hole*" OR "eighteen hole*" OR "18 hole*" OR "simulated play" OR "simulated round*" OR "driving range" (T) OR (A)
"Challenge Tour" OR "DP World Tour" OR "European Tour" OR "PGA" OR "PGA TOUR" OR NCAA OR "Ladies European Tour" OR "LET" OR "Ladies Professional Golf Association" OR LPGA OR "Middle Aged Men" (T) OR (A)
"Amateur*" OR "Professional*" OR "elite amateur*" OR "semi professional*" or "recreational golf*"
nutrition* OR diet* OR food* OR "sport* nutrition*" (T) OR (A)
"carry bag*" OR "electric trolley*" OR "push trolley*" OR "golf cart*" OR "golf bugg*" OR "single strap bag*" OR "double strap bag*" OR "golf bag*" (T) OR (A)
"energy expenditure*" OR "activity energy expenditure*" OR "energy intake" OR energy OR "caloric cost" OR "energy cost" OR "metabolic cost" OR calorie* OR "metabolic expenditure" (T) OR (A)
Caffeine* OR creatine OR supplement* (T) OR (A)
carbohydrate* OR protein* OR fat* OR macronutrient* OR micronutrient* OR vitamin*
Performance* OR "Golf* Performance*" (T) OR (A)
"putting accuracy*" OR "putting performance" OR putting OR "clubhead speed*" OR "swing speed*" OR "clubhead velocity" OR "drive distance" OR "driving accuracy" OR "shot precision*" OR "shot accuracy" (T) OR (A)
glucose* OR "energy gel*" OR gel* OR "isotonic gel*" (T) OR (A)
fluid* OR electrolyte* (T) OR (A)
hydration OR dehydration OR euhydration (T) OR (A)
"sweat rate*" OR sweat (T) OR (A)
cardiorespiatory OR cardiovascular (T) OR (A)
"cognitive function" OR "decision making" OR "cognitive-motor performance" (T) OR (A)
"Heart Rate Monitor*" OR "Actiheart Monitor*" OR "Actiheart*" (T) OR (A)
"Heart rate*" OR "Heart Beat*" (T) OR (A)
"body composition*" OR "weight" OR "lean muscle mass" OR "fat free mass" OR "adipose tissue"
"resting metabolic rate" OR RMR OR "basal metabolic rate" OR BMR (T) OR (A)
"Physiological demand*" OR "metabolic demand*" OR physiologic* (T) OR (A)
physiology OR physiologic* (T) OR (A)
"caloric expenditure"
"dietary intake*" OR "energy intake" OR "calorie intake" (T/A)
"energy availability" OR "Low energy availability" OR LEA (T/A)
"golf course" OR "golf terrain" (T/A)
